# Flying blind? Recommendations for monitoring of 10 common chronic diseases in guidelines from Germany, England, and Europe — a modified systematic review

**DOI:** 10.1186/s12916-025-04507-y

**Published:** 2025-12-01

**Authors:** Victoria Koschemann, Lisette Warkentin, Annika Viniol, Thomas Kühlein, Susann Hueber, Felix Werner

**Affiliations:** 1 Institute of General Practice, University Hospital Erlangen, Friedrich-Alexander-Universität Erlangen-Nürnberg (FAU), Universitätsstraße 29, Erlangen, Germany; 2 Institute of General Practice, University Hospital of Giessen and Marburg, Philipps University of Marburg, Karl-Von-Frisch-Straße 4, Marburg, Germany

**Keywords:** Evidence-based medicine, Clinical practice guidelines, Monitoring, Medical overuse

## Abstract

**Background:**

Clinical practice guidelines (CPGs) synthesize evidence to recommend which measures should or should not be taken in specific medical situations and thereby inform and shape the practice of medicine. In the context of an ageing population, monitoring has become an increasingly resource-intensive practice, underscoring the need for clear, evidence-based guidance. Accordingly, this study aimed to investigate the level of detail as well as the level of evidence of recommendations on monitoring for 10 common chronic diseases in CPGs from the Association of the Scientific Medical Societies in Germany (AWMF*)*, the National Institute for Health and Care Excellence (NICE), and European medical societies.

**Methods:**

Using a modified systematic review approach, a search of the relevant CPGs was conducted using the databases of the AWMF, NICE, and the Guidelines International Network (GIN). AWMF-equivalent evidence quality levels S2e and S3 CPGs were included. Recommendations on monitoring were extracted and evaluated, focusing on their level of detail regarding monitoring frequency, parameters, and consequences and their level of evidence, respectively.

**Results:**

A total of 29 CPGs were reviewed, and 163 recommendations on monitoring were extracted and evaluated. Recommendations provided a low level of detail regarding monitoring frequency in 34.4% of cases (*n* = 56), regarding parameters in 25.2% of cases (*n* = 41), and regarding consequences in 84.7% of cases (*n* = 138). A level of evidence was reported for 87 of 163 recommendations only, and if available, it was often low. Only a small proportion (6.7%; *n* = 11) of recommendations were formulated negatively as ‘do-not’ recommendations.

**Conclusions:**

The frequent lack of evidence or low level of evidence for monitoring recommendations, as well as the lack of detail in these recommendations, may lead to medical underuse, yet also to overuse, causing uncertainty among physicians and unnecessary diagnostic cascades. This has significant implications for patient harm, as well as financial and personnel burdens on the healthcare system. One potential solution could be the implementation of more ‘do-not’ recommendations in CPGs. However, overcoming systemic barriers is essential to enable the generation of high-quality evidence on monitoring.

**Supplementary Information:**

The online version contains supplementary material available at 10.1186/s12916-025-04507-y.

## Background

Chronic diseases impose a considerable burden on healthcare systems worldwide, intensifying clinical workload and driving up healthcare expenditures [1–4]. This challenge is amplified by an ageing population, leading to a further increase in chronic disease and multimorbidity [5, 6], thereby exacerbating the strain on healthcare resources. In 2020, over 60% of German patients 65 years or older reported a chronic disease or long-term health problem, with increases in prevalence starting from mid adulthood [7]. This trend is likely to continue, with far-reaching implications for healthcare systems, providers, and patients themselves [2, 8].

Addressing these challenges, secondary and tertiary prevention aim to mitigate progression of chronic diseases and to prevent complications through early detection of deterioration. Monitoring, meaning periodic assessment aimed to detect changes in disease status, plays a crucial role in this process, as it holds the promise of enabling timely intervention to improve patient outcomes or reduce complications, hospitalizations, or mortality. Yet, it remains a substantial question what and when to monitor, as well as when and how to adjust treatment [9]. If chosen inadequately, monitoring may result in overdiagnosis and overtreatment, increasing costs and workload, and imposing unneeded tests or treatments and mental burdens on patients, ultimately endangering quality of care or of life [10]. Therefore, optimal strategies for effective monitoring remain a subject of ongoing discourse.

Several clinical practice guidelines (CPGs) provide recommendations on aspects of monitoring such as frequency, parameters, and consequences. These recommendations are derived from trials, studies, or expert consensus and are intended to provide the best available external evidence as one aspect of medical decision-making in the framework of evidence-based medicine [11]. Therefore, CPGs serve as essential tools for informing medical practice and shaping patient care strategies [12]. However, the quality of evidence for recommendations varies considerably, with some based on high-quality trials and others relying on lower-tier evidence such as observational studies or expert consensus.

To date, no systematic review has examined the evidence base of recommendations on monitoring. Yet, understanding the evidence base underpinning CPG recommendations on monitoring is primarily crucial for medical decision-making, but also for highlighting the potential need for more robust evidence as well as for increasing research funding, and especially for appropriately weighting evidence within the framework of evidence-based medicine. This is particularly important in the context of chronic disease management, where the consequences of suboptimal as well as excessive monitoring can be severe. Thus, the primary objective of the present study was to identify recommendations concerning the monitoring of common chronic diseases prevalent in general practice, outlined in CPGs from the German Association of the Scientific Medical Societies (Arbeitsgemeinschaft der wissenschaftlichen medizinischen Fachgesellschaften — *AWMF*), from the National Institute for Health and Care Excellence (*NICE*) for England and Wales, and from European medical societies. We further aimed to elucidate their level of detail, strength of recommendation (SoR), and their level of evidence (LoE).

## Methods

To examine recommendations on monitoring for 10 prevalent chronic diseases in general practice, we conducted a stratified search based on a modified systematic review approach: First, relevant AWMF, NICE, and European medical societies’ CPGs of sufficient quality levels were identified, before they were reviewed to ascertain recommendations on monitoring. Reporting of the study is based on the PRISMA (Preferred Reporting Items for Systematic reviews and Meta-Analyses) recommendations (see Additional file 1: Table S1). As a review of CPGs, registration of the protocol on PROSPERO was not eligible. This study was part of the project ChroMO — Monitoring Routines for People with Chronic Conditions: A Baseline Assessment and Roadmap for the Future, funded by the Innovation Fund of the German Federal Joint Committee (G-BA) (grant number 01VSF22046).

### Identification of chronic diseases

For this investigation, 10 chronic diseases considered relevant — based on informal consensus among participating researchers in the field of general practice and selected because of their high prevalence in general practice — were identified: coronary heart disease (CHD), chronic heart failure (CHF), diabetes mellitus type 2 (DM2), bronchial asthma, chronic obstructive pulmonary disease (COPD), depression, hypothyroidism, chronic kidney disease (CKD), ischaemic stroke, and osteoporosis. The prevalences of these conditions in Europe vary, ranging from approximately 2% for stroke [13] to 5.1% and 5.6% for asthma [14] and osteoporosis [15], respectively, and reaching up to almost 12% for chronic kidney disease [16].

### Search strategy

The literature screening was conducted in November and December 2023. National CPGs were retrieved from the AWMF registry in Germany and the NICE registry in England and Wales via manual search. In the case of the AWMF, the search was restricted to CPGs of the highest quality levels S2e and S3. To qualify for quality level S2e, CPGs have to be supported by a systematic evidence base, which is required to be evaluated for its strengths and weaknesses, while S3 CPGs additionally include structured consensus processes among experts and stakeholders [17]. The search in the NICE registry was limited to National Guidelines (NG) and Clinical Guidelines (CG). Due to the unavailability of centralized registers for European medical societies’ CPGs, the Guidelines International Network (*GIN*) database was used in a forward–backward search manner to first identify relevant European medical societies. Additional file 2: Table S2 contains the search strings used. Following their identification, a manual search was conducted for relevant CPGs authored by these societies. Subsequently, titles and, if available, abstracts were screened.

### Inclusion and exclusion criteria

CPGs were considered for evaluation of recommendations if they addressed 1 of the 10 diseases and were valid at the time of search. CPGs were successively excluded if they did not address 1 of the 10 relevant diseases or if they focused solely on acute care settings or paediatric populations. If considered relevant, NICE and European medical societies’ CPGs were evaluated in regard to their overall quality of evidence; AWMF CPGs were not evaluated as the search was conducted for quality level S2e and S3.

### Evaluation of CPG quality

CPGs not found in the AWMF registry were assessed for allocation to an overall quality level using the Appraisal of Guidelines for Research and Evaluation (AGREE) II tool. This approach was chosen because certain items represent the three key characteristics of a higher quality of evidence: interdisciplinary group with patient representatives, systematic search for evidence, and formal consensus-building [17]. If items 7, 8, and 9 of domain 3 of AGREE II scored at least 5 points, quality level S2e was awarded. In addition, if items 4, 5, and 10 of domains 2 and 3 were rated at least 5 points, quality level S3 was awarded (cf. Table 1). The assessment was performed by two assessors (V. K., F. W.) independently and blinded. Differences of more than 1 point were resolved by discussion and agreement between the assessors after unblinding. If an item had an average score of 4.5, the score was rounded up. If one or both assessors scored 1 point under the threshold for S2e or S3 level in a maximum of two items, the corresponding CPG was discussed again after unblinding to reach a consensus.

**Table 1:** Overview of the AGREE II Items evaluated for stratification into AWMF evidence levels (Hoffmann-Eßer W. et al. *Guideline appraisal with AGREE II: systematic review of the current evidence on how users handle the two overall assessments*, PLOS ONE 2017)

AWMF evidence level	AGREE II domain	AGREE II item	Leading aspect
S2e	3	7	Systematic methods were used to search for evidence
	3	8	The criteria for selecting the evidence are clearly described
	3	9	The strengths and limitations of the body of evidence are clearly described
S3	2	4	The guideline development group includes individuals from all relevant professional groups
	2	5	The views and preferences of the target population (patients, public, etc.) have been sought
	3	10	The methods for formulating the recommendations are clearly described

### Evaluation of recommendation quality

All recommendations in the included CPGs were assessed on their relevance for monitoring as well as for the general practice setting by one of two assessors (L. W., F. W.). If an assessor was uncertain about inclusion, both assessors discussed the recommendation and agreed on its inclusion or exclusion by consensus.

The recommendations were evaluated in terms of the level of detail provided for the parameters to be measured, the monitoring intervals, and any consequences arising from the results of monitoring that should be taken by two assessors (V. K., F. W.). A three-tiered assessment framework was employed, which categorized the level of detail as either low (no or minimal level of detail, e.g. ‘Monitor side effects’ in regards to parameters), moderate (medium level of detail, e.g. ‘Monitor liver function’), or high (high level of detail, e.g. ‘Monitor liver transaminases’).

Additionally, for each recommendation, the LoE and the SoR were assessed. Where applicable, both ratings were taken directly from the CPGs. In instances where a recommendation referred to an earlier CPG version with regard to LoE, it was adapted from that version. This approach resulted in different LoE classifications being assigned within the same CPG in some cases. In these cases, all classifications were analysed. Previous CPG versions that were no longer available through standard access routes were retrieved, where possible, from the *Internet Archive Wayback Machine* (https://web.archive.org).

Recommendations that were either stated as consensus based or that referred only to indirect evidence or evidence of unclear directness that could not be substantiated by further research were assigned ‘no direct or unclear evidence’. This applies as well to instances where CPGs stated that they had not conducted their own evidence grading but had taken it from other documents, or if they cited evidence, but no evidence grading could be identified.

SoR was assigned to AWMF recommendations according to official nomenclature (A: should (not), B: ought (not), 0: may be considered/omitted) [17]. NICE recommendations were assigned an indicator of SoR based on their wording according to the published manual (1: must (not), 2: should (not), 3: could) [18].

Furthermore, the direction of the recommendation was specified in order to differentiate between positive and negative recommendations (‘do-not’ recommendations).

## Results

### Search results

**Fig. 1:**
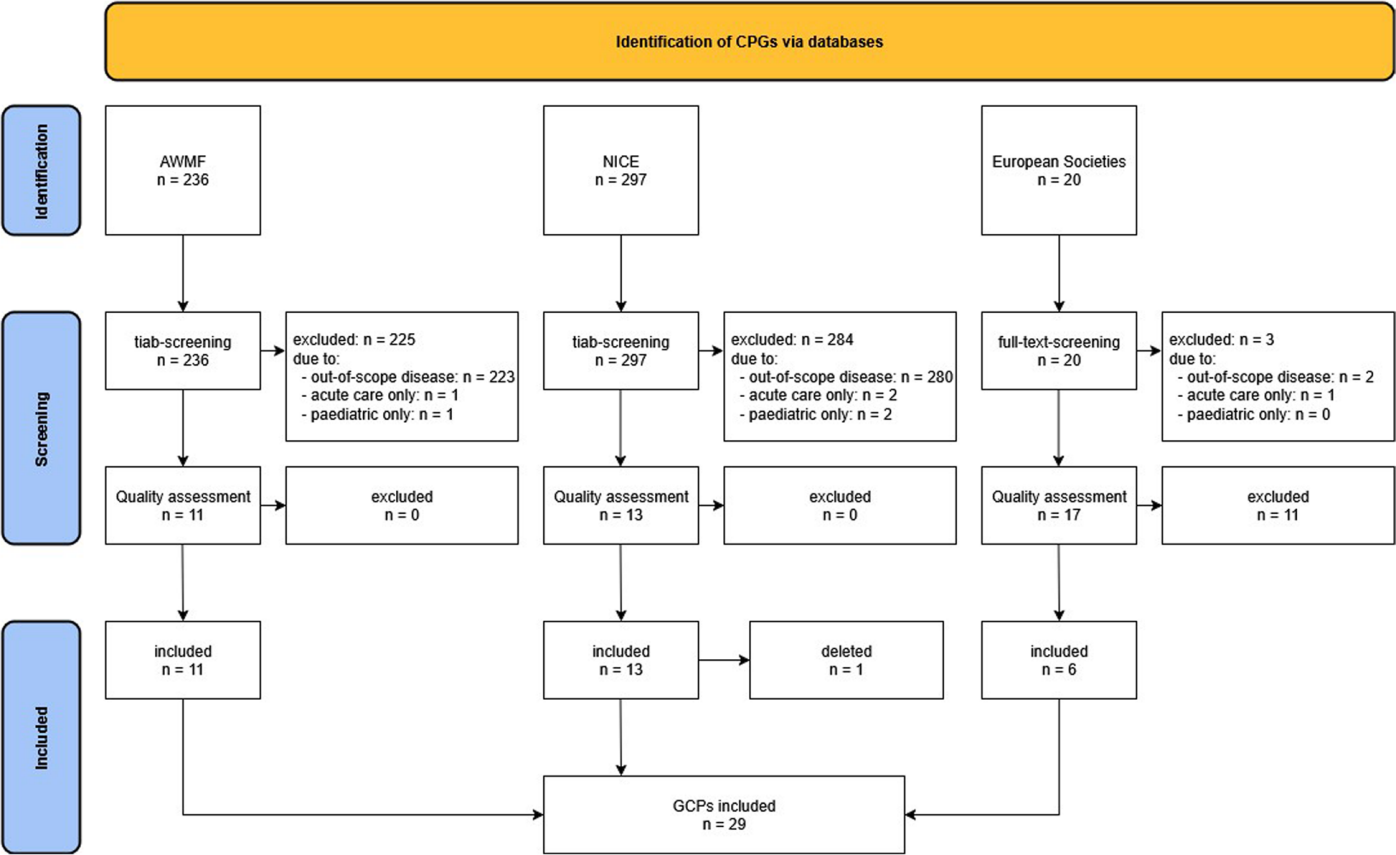
PRISMA flow chart

The AWMF search yielded a total of 236 guidelines, while 297 results were obtained from NICE. Following the screening of the results, 509 CPGs were excluded, leaving 11 and 13 CPGs from AWMF and NICE, respectively [19–42]. None of the CPGs was removed due to quality reasons.^1^ The GIN-based forward–backward search yielded a total of 20 CPGs [43–62] from European medical societies, 14 of which were excluded during full-text evaluation due to not meeting the inclusion criteria, dealing with other than the 10 diseases or in an acute setting [43, 44, 57], or poor AGREE-II quality rating mainly due to insufficient evidence search [45, 48, 50–52, 54–56, 58, 61, 62]. Cohen’s *κ* for AGREE-II quality rating was 0.59. In conclusion, we identified 30 CPGs that were searched for recommendations on monitoring [19–42, 46, 47, 49, 53, 59, 60]. One of the NICE CPGs included in the initial search [31] was deleted and incorporated into other CPGs in May 2024 during the subsequent stages of the search process. Another CPG was classified as S3 level, yet the chapter addressing monitoring was only S2k level, so the recommendations were included in this study as consensus based [29] (Fig. 1).

All 29 of CPGs were then subjected to an evaluation of their recommendations on monitoring. Six CPGs, all of them from European medical societies, did not contain any recommendations on monitoring [46, 47, 49, 53, 59, 60]. The remaining 23 AWMF and NICE CPGs contained a total of 2544 recommendations that were subjected to review, and 163 of these were deemed to bear relevance to monitoring (per CPG: median = 6, range = 1–17). Recommendations for monitoring in both NICE and AWMF CPGs were identifiable for 9 of the 10 selected chronic diseases; no AWMF CPG that met the predefined inclusion criteria was available for hypothyroidism. Regarding European medical societies’ CPGs, no relevant CPG for CKD was identified. Additional file 3: Table S3 contains all CPGs with their correspondent ratings.

### Level of detail

A notable variation in the level of detail was observed across the recommendations. With regard to the parameters to be collected as part of the monitoring measure, 25.2% (*n* = 41) of the recommendations provided low-level detail, 19.6% (*n* = 32) provided mid-level detail, and 49.2% (*n* = 90) provided high-level detail. In terms of the frequency of monitoring dates, 34.4% (*n* = 56) of the recommendations provided low-level detail, 27.6% (*n* = 45) provided mid-level detail, and 38.0% (*n* = 62) provided high-level detail. The most significant disparity was observed in the consequences of monitoring: the majority of the recommendations scarcely addressed these consequences (84.7%, *n* = 138); only a minority of the recommendations provided mid-level (7.4%, *n* = 12) or high-level detail (8.0%, *n* = 13).

### Level of evidence of recommendations on monitoring

Following a thorough review, it was determined that 87 out of 163 recommendations analysed had no identifiable primary evidence, including five evidence statements from the British National Formulary that were inaccessible to the reviewers. For the remaining 76 recommendations, a total of 135 evidence statements providing at least partial evidence were identified. In instances where primary evidence was identified, the classifications for LoE included six evidence grading systems. Table 2 shows the identified evidence grading systems and their corresponding levels.

**Table 2:** Used evidence grading systems with corresponding level

Evidence grading system	Levels of evidence grading system (lowest to highest)
GRADE [63]	Very low (ØOOO), low (ØØOO), medium (ØØØO), high (ØØØØ)
CEBM 2009 [64]	5, 4, 3b, 3a, 2c, 2b, 2a, 1c, 1b, 1a^a^
CEBM 2011 [65]	5, 4, 3, 2, 1
AHCPR [66]	4, 3, 2b, 2a, 1b, 1a
SIGN [67]	4, 3, 2, 2+, 2++, 1−, 1+, 1++
ACCP [68]	C, B, A

^a^One of the CPGs used an evidence grading system based on CEBM 2009 but with only six levels (V, IV, III, II, Ib, Ia) [26]

**Fig. 2:**
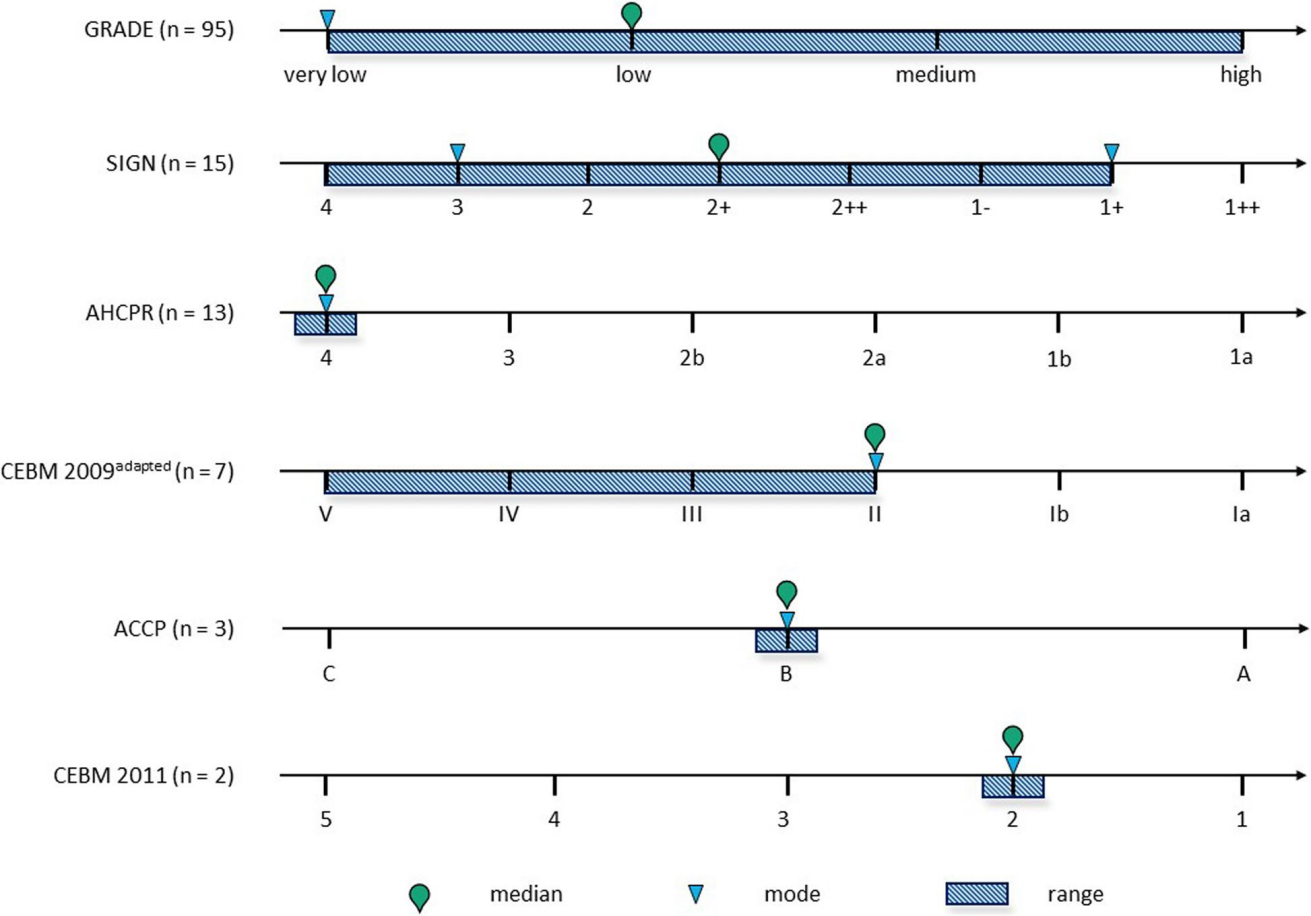
Distribution of levels of evidence of those recommendations with identifiable evidence (*n* = 135) from lowest level of evidence on the left to highest level of evidence on the right. The blue bar represents the minimum, maximum, and range, the red triangle indicates the mode, and the red pin indicates the median of levels of evidence

When direct evidence was identified, it rarely reached the highest LoE. The most widely used evidence grading system was GRADE, which was applied in 95 out of 135 instances, with the majority of evidence levels being classified as very low (mode) and low (median). The second most common evidence grading system was SIGN, used in 15 instances, with a bimodal distribution at levels 3 and 1 + and a median level of 2 +. This was followed by AHCPR, used in 13 instances, with a mode and median level of 4, and the adapted CEBM 2009, used in 7 instances, with a mode and median level of II. In contrast, the least frequently used grading systems were ACCP, used in three instances, with a mode and median level of B, and CEBM 2011, used in two instances, with a mode and median level of 2. Figure 2 shows the distribution of evidence gradings. Additional file 4: Table S4 contains the single recommendations identified, their level of detail, LoE, and SoR; German recommendations have been translated with special consideration of the congruence of wording to their SoR to avoid misconceptions. Additional file 5: Table S5 contains the level of detail and LoE per CPG, per provenance, and overall.

### Strength of recommendation

**Fig. 3:**
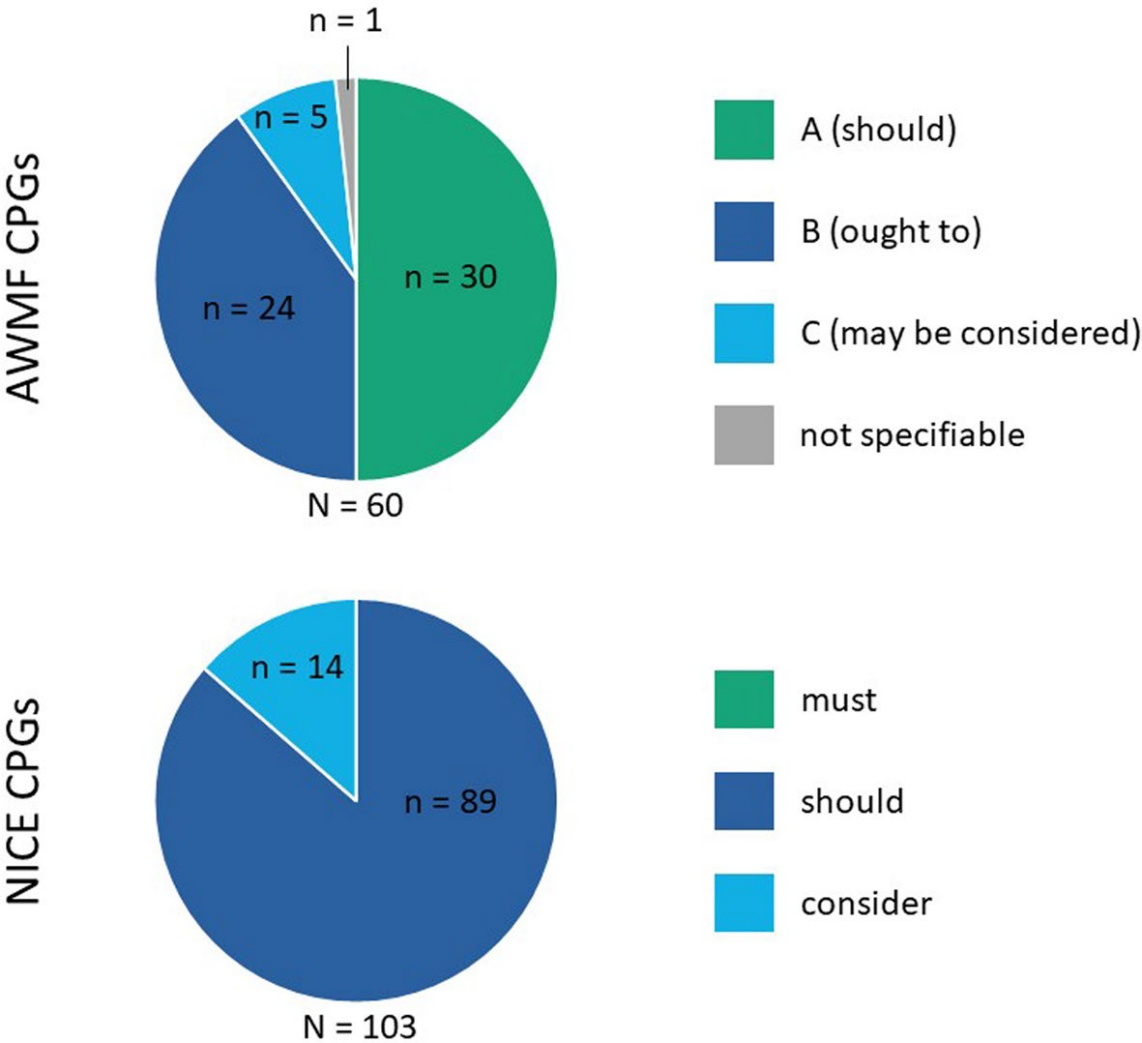
Distribution of strength of recommendation (*n* = 163) in AWMF CPGs (top) and NICE CPGs (bottom)

Both the AWMF and NICE CPGs employ three-tier systems for SoR, albeit with distinct terminology. The AWMF CPGs’ recommendations (*n* = 60) utilized the nomenclature ‘should’ (*n* = 30), ‘ought to’ (*n* = 24), and ‘may be considered’ (*n* = 5); however, one recommendation lacked an assignable SoR due to ambiguous wording. In contrast, the examination of the NICE CPGs (*n* = 103) revealed a different distribution, with no recommendations classified as ‘must’, *n* = 89 as ‘should’, and *n* = 14 as ‘consider to’ (Fig. 3). If the assessment was limited to recommendations for which no direct evidence was identified (*n* = 80), we observed the following distribution: *n* = 20 ‘should’, *n* = 14 ‘ought to’, and *n* = 2 ‘may be considered’ recommendations (AWMF) and *n* = 0 ‘must’, *n* = 39 ‘should’, and *n* = 10 ‘consider’ recommendations (NICE), respectively.

### Negative recommendations

Among the 163 analysed recommendations, 6.7% (*n* = 11) were exclusively negative, advising against a particular action (‘do not’, ‘consider not to’), while 2.5% (*n* = 4) contained both positive and negative aspects. Overall, 9.2% (*n* = 15) of the recommendations were at least partially negative. In contrast, the vast majority of recommendations (90.8%, *n* = 148) were solely positive, advocating for a specific action (‘do’, ‘consider to do’).

## Discussion

In a comprehensive analysis of 29 CPGs, 163 recommendations on monitoring were identified. Only half of these recommendations provided high-level detail on monitoring parameters, and nearly 4 in 10 recommendations provided high-level detail regarding monitoring frequency. Furthermore, the consequences of monitoring were only adequately described in less than 20% of recommendations. Eighty-seven of the 163 recommendations lacked an identifiable evidence base, while the remaining recommendations had 135 evidence specifications that could be identified, mostly with very low or low LoE according to the GRADE system, the most frequently used evidence grading system. Despite the widely low LoE, the SoR reached moderate to high levels in most recommendations. Moreover, the vast majority of recommendations were positive, recommending for instead of against specific actions.

More than half of the recommendations on monitoring in these CPGs were based on no or no identifiable evidence; where direct evidence was identified, the majority of recommendations relied on low evidence levels. A similar result was reached by a study from the UK in 2019 analysing a smaller number of CPGs. Monitoring strategies for patients with type 2 diabetes, CKD, and hypertension were reviewed, including six English CPGs. The authors concluded that UK CPGs for chronic disease monitoring in primary care were largely based on expert opinion, with robust evidence for optimal monitoring strategies lacking [10]. This aligns with our findings, highlighting the widespread absence of high-quality evidence in CPG recommendations on monitoring. This lack of solid evidence gives rise to considerable uncertainty regarding both the efficacy and the optimal methods of monitoring, thereby fostering a proliferation of heterogeneous monitoring approaches. This does not apply exclusively to the practice of monitoring, as research has demonstrated that in surgery, even the best available evidence is often of low quality [69]. At the same time, the fact that even strong recommendations may rest on limited evidence may evoke the assumption that the presumed indispensability of high-quality data is open to question, and that the value of high-quality studies in evidence-based medicine could be undermined. Lastly, this may lead to an erosion of confidence in CPGs and thus compromise consistency of patient care. It is therefore of utmost importance to emphasize the continued generation of the highest possible quality of evidence.

In some cases, it was not possible to fully distinguish between a substantial lack of evidence and a lack of accessibility of that evidence. In the case of ‘deep’ cross-references, we also tried to access older versions of CPGs. However, the evidence was often lost in older documents, or the source documents could no longer be found. This highlights an underlying problem of a growing body of evidence in evidence-based medicine, namely that ‘evidence narratives’ develop over time which, at least superficially, are no longer scrutinized — and may no longer be questioned because their origin is hardly accessible or lost in the background noise of cross-reference. In addition, restricted access to key reference resources, such as the British National Formulary, complicates the feasibility of systematic evidence assessments.

Building on these observations, a closer examination of the CPG recommendations revealed not only a lack of robust evidence but also a scarcity of detailed information to guide clinical practice. Specifically, our study highlights insufficient detail in CPG recommendations. Most of them provided sufficient information on monitoring parameters but omitted recommending consequences of monitoring or specific guidance on monitoring frequency. This lack of detailed statements on monitoring frequency is in line with prior research, where the recommended frequency of testing varied between CPGs or was entirely lacking, and the recommendations on monitoring frequency were based on expert opinion [10]. Similar to the general lack of monitoring recommendations in CPGs, as already mentioned, the absence of clear specifications regarding monitoring frequencies may lead to undertreatment if measures are omitted. However, the contribution of lacking recommendations or sufficient levels of detail to an uncontrolled proliferation of measures, particularly against the background of diagnostic uncertainty or defensive medicine, may be considered equally significant [70]: it may contribute to unnecessary testing of patients with chronic diseases, leading to increased workload and health costs [71, 72]. Evidence from the UK demonstrated that excluding liver function tests and full blood counts from routine monitoring for several conditions was associated with a reduction of liver function tests by 14% and of full blood counts by 22%, resulting in estimated savings of GBP 200,000 in a population of 180,000 patients, without an observed reduction in the identification of potentially significant disease [71]. When extrapolated to the national level, such measures may translate into substantial savings in healthcare expenditure. On the patients’ side, unnecessary testing may contribute to patient anxiety or even mistrust in the medical system and place additional demands on their time resources. Perhaps most importantly, however — relevant for both the health system and for patients — it carries the risk of overdiagnosis, with the potential for false positives, subsequently leading to unnecessary examinations and to diagnostic or therapeutic cascades. This is exemplified in cholesterol monitoring, where the low signal-to-noise ratio [73] renders a substantial proportion of testing potentially unnecessary [74], and subsequent reactions to these measurements may be potentially incorrect. Additionally, inappropriate reactions to test results may also result in unnecessary or even harmful adjustments to treatment. A UK study on monitoring digoxin serum levels showed that 82.5% of test requests lacked clear clinical justification, with inappropriate decisions in one-fourth of cases [75], while a 2003 New Zealand study found that 53% of ordered measurements were timed inappropriately and 5% of ordered measurements led to inappropriate dose adjustments [76].

Notably, the assignment of moderate to high levels of SoR to recommendations lacking direct evidence is a striking phenomenon. This observation suggests that the SoR is not closely tied to the underlying evidence base, which in turn implies that the safety and validity of the recommendations may not be adequately considered. This divergence has already been described for CPGs from cardiology societies [77]. However, the clinical importance of different monitoring schemes in chronic diseases may vary considerably, depending on disease progression and the potential consequences of delayed detection. In this context, it may be reasonable to consider that the degree of clinical relevance might, in certain cases, justify a stronger recommendation, even if the underlying evidence is limited.

Another notable finding our study reveals is that the vast majority of recommendations (90.8%) were positive, advising for the implementation of a particular measure. In contrast, only 9.2% of the analysed recommendations were at least partially negative. This stands in contrast with the findings of a German survey, in which 88% of general practitioners expressed a desire for greater emphasis on the avoidance of certain procedures, highlighting ‘do-not’ recommendations, and for a reduction of healthcare overuse in CPGs [78]. This is particularly relevant as uncritical adherence to positive CPG recommendations may increase the risk of polypharmacy and medication interactions in patients with multiple chronic conditions [79], while negative recommendations may have the potential to reduce such risks. In light of the limited evidence base underpinning recommendations on monitoring, it may be beneficial to consider incorporating more negative recommendations to mitigate overuse without substantially increasing the risk of underuse. A corresponding observation was reported in a US study, where approximately half of the physicians surveyed viewed the use of CPGs as a means of reducing overtreatment [80].

The deficit in the details on frequency in CPG recommendations might partially be rooted in a paucity of scientific studies addressing optimal monitoring strategies of chronic diseases. Current research focuses on the evaluation of diagnostic and prognostic accuracy of tests, rather than on defining optimal testing frequency [81], with a few exceptions, especially with regard to HbA_1c_ testing [82–86]. To address these uncertainties and provide evidence, future studies are essential. Yet, using randomized controlled trials to provide the highest evidence levels for the evaluation of frequencies poses ethical challenges — randomizing a control group to a lower frequency of monitoring or to prolonged periods without any monitoring might raise ethical concerns. Moreover, evaluations of monitoring regimens for chronic diseases typically necessitate extended follow-up periods or very large cohorts to detect modest outcome differences. These requirements are not adequately addressed in current calls for proposals and funding programmes, which are dependent on time-limited public funds or sponsors from industry with narrowly defined interests. This challenge might be alleviated through dedicated funding schemes specifically designed for long‐term research, such as registry‐based studies. Such specialized grants could underwrite the extended follow‐up and large‐scale data collection that conventional, time-limited funding mechanisms often cannot support. An alternative approach could involve observational studies comparing regions with low versus high monitoring practices yet require careful consideration of sources of bias. To improve accessibility and comparability of CPGs, harmonizing minimum standards for guideline development and establishing central registries for quality control might be regarded as a possible approach.

### Limitations

To our knowledge, this is one of the first comprehensive studies on recommendations on monitoring and their evidence base. However, different limitations remain.

First, given the absence of a central registry for CPGs from European medical societies, we employed GIN to identify pertinent societies, which were subsequently searched for relevant CPGs. While this strategy enabled a systematic and efficient identification of potential sources, it also relied on a general completeness of GIN and the individual societies’ websites. It is possible that European medical societies not indexed in GIN have published CPGs, which subsequently were unable to be considered. Since no additional database search (e.g. in MEDBASE or Embase) was conducted, this may have introduced a selection bias, as CPGs disseminated through alternative platforms could have been overlooked. Nevertheless, since CPGs from European specialist organizations could be identified for 9 of the 10 diseases, with the exception of CKD (for which the ERA-EDTA would be the responsible medical specialist organization but only refers to the international CPGs of the KDIGO), a high degree of saturation of our search can be assumed.

Second, the analysis of the evidence base for monitoring recommendations is subject to a notable selection effect, owing to the complex research-practical and ethical requirements inherent in studies on monitoring regimens, as mentioned above. Consequently, this analysis cannot be considered representative of the entire evidence base of CPG recommendations. In contrast to recommendations concerning the treatment of chronic diseases, which are often informed by randomized controlled trials and thereby achieve higher LoE, the evidence base for monitoring recommendations is likely to be informed by observational studies, achieving lower LoE. It is therefore reasonable to assume that the evidence ratings for monitoring recommendations are, on average, lower than those for diagnostic or therapeutic recommendations. Furthermore, because the study included CPGs only from selected European countries and medical societies, its findings cannot be readily extrapolated to other global contexts.

Finally, this work may be subject to bias due to the retrospective nature of data sourcing. Older versions of CPGs were consulted whenever current versions referred to earlier evidence ratings, using a digital archive of former website versions. In some cases, however, access to earlier CPGs was not possible, which may have introduced bias. Yet, precisely this limitation reinforces the interpretation of mentioned ‘evidence narratives’: if the origins of evidence are no longer verifiable, the fragility of evidence becomes apparent, and its influence must be critically acknowledged.

## Conclusions

For an evidence-based practice, recommendations based on high-quality external evidence are a crucial component. Our study highlights the lack of robust LoE underlying the majority of recommendations on the monitoring of common chronic diseases in AWMF and NICE CPGs. Given the limitations of current evidence, it is imperative to generate new evidence through studies and evaluation research, thereby improving the quality and accuracy of monitoring recommendations and patient care. Yet, the absence of high-level empirical evidence to support many recommendations on monitoring does not ultimately invalidate the utility or efficacy of these specific recommendations, as — in the context of evidence-based medicine — even expert consensus can serve as a basis for medical decision-making if it represents the best available evidence [11]. Abstaining from issuing recommendations on account of insufficient evidence risks fostering an unchecked proliferation of interventions. Until more robust evidence is generated, clinical practitioners must accept and manage this contradiction in evidence-based medicine — there is an urgent need for clinicians to provide patients with information about the potential harms of unnecessary testing, facilitating informed decision-making, and for CPG developers to ensure that recommendations consider the risks of monitoring as well.

## Supplementary Information

Additional file 1. Table S1 – PRISMA Checklist.

Additional file 2. Table S2 – Guidelines International Network (GIN) registry search strings.

Additional file 3. Table S3 – Summary table of all CPGs and correspondent ratings.

Additional file 4. Table S4 – Summary table of identified recommendations and assessment ratings.

Additional file 5. Table S5 – Summary table of assessment ratings at CPG level, provenance, and overall.

## Data Availability

Data that support the findings of this study are included in the supplemental files of this article or, if connected to individual researchers, available from the corresponding author for researchers who provide a methodologically sound proposal.

## Footnotes

1 As the search within the AWMF network was restricted to S2e and S3 CPGs, the categorization of the CPGs was adopted.
