# Supplementary material for: Flying blind? Recommendations for monitoring of 10 common chronic diseases in guidelines from Germany, England, and Europe — a modified systematic review
Source: BMC Med. 2025 Dec 1;23:671. doi: 10.1186/s12916-025-04507-y (PMC12670874; doi:10.1186/s12916-025-04507-y)
Supplement: Supplementary file 2 — Additional file 2. Table S2 – Guidelines International Network (GIN) registry search strings. [file 12916_2025_4507_MOESM2_ESM.docx]

**Supplementary File 2. Search strings used in the *Guidelines International Network* (GIN) registry**

| **disease** | **search strings** |
| --- | --- |
| coronary heart disease | „coronary heart disease“, „coronary artery disease“, „CAD“, „coronary disease“, „atherosclerotic heart disease“ |
| chronic heart failure | „chronic heart failure“, „heart failure“, „CHF“, „chronic cardiac insufficiency“ |
| diabetes mellitus type 2 | „diabetes mellitus type 2“, „diabetes mellitus“, „diabetes“, „type 2 diabetes“, „T2D“ |
| bronchial asthma | „bronchial asthma“, „asthma“, „allergic asthma“ |
| chronic obstructive pulmonary disease | „chronic obstructive pulmonary disease“, „COPD“, „obstructive lung disease“ |
| depression | „depression“, „major depressive disorder“, „unipolar depression“ |
| hypothyroidism | „hypothyroidism“, „thyroid deficiency“ |
| chronic kidney disease | „chronic kidney disease“, „CKD“, „chronic kidney failure“, „chronic renal insufficiency“ |
| ischemic stroke | „stroke“, „ischemic stroke“, „cerebral infarction“, „brain ischemia“ |
| osteoporosis | „osteoporosis“ |
